# Epigenetic histone H3 phosphorylation marks discriminate between univalent- and bivalent-forming chromosomes during canina asymmetrical meiosis

**DOI:** 10.1093/aob/mcad198

**Published:** 2023-12-21

**Authors:** Radka Kalfusová, Veit Herklotz, Katrin Kumke, Andreas Houben, Aleš Kovařík, Christiane M Ritz, Jana Lunerová

**Affiliations:** Institute of Biophysics of the Czech Academy of Sciences, Královopolská 135, 612 00 Brno, Czech Republic; Senckenberg Museum of Natural History, Senckenberg – Member of the Leibniz Association, Am Museum 1, 02826 Görlitz, Germany; Leibniz Institute of Plant Genetics and Crop Plant Research (IPK), Gatersleben, 06466 Stadt Seeland, Germany; Leibniz Institute of Plant Genetics and Crop Plant Research (IPK), Gatersleben, 06466 Stadt Seeland, Germany; Institute of Biophysics of the Czech Academy of Sciences, Královopolská 135, 612 00 Brno, Czech Republic; Senckenberg Museum of Natural History, Senckenberg – Member of the Leibniz Association, Am Museum 1, 02826 Görlitz, Germany; Chair of Biodiversity of Higher Plants, Technical University Dresden, D-01069, Dresden, Germany; Institute of Biophysics of the Czech Academy of Sciences, Královopolská 135, 612 00 Brno, Czech Republic

**Keywords:** *Rosa* genus, dogroses, canina meiosis, histone H3 phosphorylation, euchromatin and heterochromatin, 18S ribosomal DNA, non-disjunction, fluorescence in situ hybridization, immunostaining

## Abstract

**Background and Aims:**

Dogroses (*Rosa* sect. *Caninae*) are mostly pentaploid, bearing 2*n* = 5*x* = 35 chromosomes in somatic cells. They evolved a unique form of asymmetrical meiosis characterized by two types of chromosomes: (1) chromosomes forming bivalents and distributed in the normal sexual way; and (2) chromosomes occurring as univalents and transferred by a female gamete only. In the mature pollen of pentaploid species, seven bivalent-derived chromosomes are transmitted to offspring, and 21 unpaired univalent chromosomes are eliminated during microsporogenesis. To discriminate between bivalent- and univalent-forming chromosomes, we studied histone H3 phosphorylation patterns regulating meiotic chromosome condensation and segregation.

**Methods:**

We analysed histone modification patterns during male canina meiosis in two representative dogrose species, 5*x Rosa canina* and 5*x Rosa rubiginosa*, by immunohistochemical and molecular cytogenetics approaches. Immunostaining of meiotic cells included α-tubulin, histone H3 phosphorylation (H3S10p, H3S28p and H3T3p) and methylation (H3K4me3 and H3K27me3) marks. In addition, fluorescent *in situ* hybridization was carried out with an 18S rDNA probe.

**Key Results:**

In the first meiotic division, univalent chromosomes underwent equational division into chromatids, while homologues in bivalents were segregated as regular dyads. In diakinesis, bivalent chromosomes displayed strong H3 phosphorylation signals in proximal regions, spreading to the rest of the chromosome. In contrast, in univalents, the H3 phosphorylation signals were weaker, occurring mostly outside proximal regions largely overlapping with the H3K4me3 signals. Reduced phosphorylation was associated with relative under-condensation of the univalent chromosomes, particularly at early diakinesis.

**Conclusions:**

We hypothesize that the absence of pairing and/or recombination in univalent chromosomes negatively affects the histone H3 phosphorylation of their chromatin and perhaps the loading of meiotic-specific cohesins. This apparently destabilizes cohesion of sister chromatids, leading to their premature split in the first meiotic division.

## INTRODUCTION

Polyploidy is a ubiquitous phenomenon in plants, given that whole-genome duplications have been detected in all major lineages (Van De Peer *et al.*, 2017), and approximately one-quarter of vascular plant species represent recent polyploids (Barker *et al.*, 2016). An immediate challenge for newly arisen polyploids is the maintenance and stability of sexual reproduction based on effective meiotic division (Alix *et al.*, 2017). This is usually acquired in a relatively easy manner by allopolyploids, where polyploidy is coupled with hybridization, leading to selective chromosome pairing of homoeologous chromosomes (Ramsey and Schemske, 1998; Grandont *et al.*, 2013). In contrast to plants with even ploidy levels, polyploids with odd numbers of chromosomes (e.g. 2*n* = 3*x*, 5*x*, 7*x* = 21, 35, 49) probably are restricted either to vegetative reproduction or can reproduce by apomixis, avoiding meiosis completely (e.g. *Rubus* L., *Taraxacum* F.H. Wiggs) (Van Dijk, 2003). In addition, a small number of plant lineages have independently evolved intermediate hemisexual mechanisms of meiotic recombination, in which there is differentiation between recombining (bivalent-forming) and apomictically inherited (univalent-forming) chromosomes, e.g. *Rosa* sect. *Caninae* (DC.) Ser., sect. *Rubigineae* (DC.) Christ (Täckholm, 1922), *Leucopogon* R. Br. (Smith-White, 1948) and *Onosma* L. (Kolarčik *et al.*, 2014; Teppner, 1971).

**Fig. 1.**
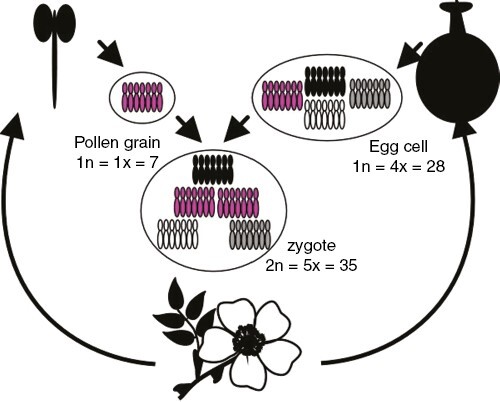
Diagram of canina meiosis, an asymmetrical form of meiosis. Dogroses with a pentaploid somatic chromosome number (2*n* = 5*x* = 35) produce haploid (1*n* = 1*x* = 7) pollen grains during microsporogenesis in the anthers and tetraploid (1*n* = 4*x* = 28) egg cells during megasporogenesis in the carpels. Figure reproduced from Ritz and Wissemann (2011) under the Creative Commons licence (https://creativecommons.org/licenses/by/2.0).

Section *Caninae* of the genus *Rosa* L. represents a highly successful group of wild roses called dogroses (~60 species in total) distributed in Europe and Western Asia (Wissemann, 2003; Koopman *et al.*, 2008). All dogroses are polyploids originating from multiple hybridization events (Wissemann, 2000; Ritz *et al.*, 2005); most species are pentaploid (2*n* = *5x* = 35), with some tetraploids, hexaploids and heptaploids (2*n* = 4*x*, 6*x*, 7*x* = 28, 42, 49) (Klášterská, 1969; Wissemann, 2002; Herklotz and Ritz, 2017). Dogroses display a peculiar form of asymmetrical meiosis called ‘canina meiosis’, which was observed more than a century ago (Täckholm, 1920; Blackburn and Harrison, 1921). Regardless of ploidy level, only 14 chromosomes pair in meiosis (seven bivalents) and are regularly distributed to the male and female gametes, while the remaining chromosomes are transmitted as univalents via the egg cell (Fig. 1). Thus, male and female gametes contribute unequally to the chromosome complement of the zygote. The resulting matroclinal inheritance of genetic material has been confirmed by microsatellite and ribosomal DNA (rDNA) markers (Nybom *et al.*, 2004, 2006; Kovarik *et al.*, 2008; Herklotz and Ritz, 2017). A population-level study involving thousands of European samples revealed considerably fewer copies of identical multi-locus genotypes in dogroses in comparison to species with regular sexual reproduction (Reichel *et al.*, 2023), supporting their considerable genetic diversity. In male meiosis, univalent chromosomes behave as laggards, i.e. display retarded migration compared with that of bivalents in the first meiotic division. The expression activity of genes located on univalent chromosomes has been debated considerably (Darlington, 1965; Khaitová *et al.*, 2010; Ritz *et al.*, 2011; Vogt *et al.*, 2015). Earlier studies suggested these chromosomes to be primarily inactive and heterochromatic (Darlington, 1965), whereas more recent studies revealed transcriptional activity of genes encoded by chromosomes forming univalents (Ritz *et al.*, 2011; Herklotz *et al.*, 2018). However, chromosome studies in roses are methodologically challenging because of the small size of the chromosomes and their sticky behaviour (Klášterská and Natarajan, 1974*a*, Kirov *et al.*, 2014, 2016; Klásterská and Natarajan, 2014) and generally low number of mitotic and meiotic cells. In addition, a high content of polyphenolic compounds in the cytoplasm complicates the preparation of microscopic samples (Roman *et al.*, 2013). Nevertheless, several classical cytogenetic studies confirmed the original findings of Täckholm (1922), showing highly polarized meiosis in dogrose spermatocytes (Klášterská and Natarajan, 1974*a*; Roberts, 1975; Lata, 1982; Lim *et al.*, 2005).

The epigenetic control of the cell cycle through post-translational histone modifications (phosphorylation, acetylation, methylation, etc.) plays a fundamental role directly related to changes in chromatin structure and function. Phosphorylation of a serine residue of the H3 histone at position 10 (H3S10p) is the most common histone modification with a direct link to meiosis and mitosis in both plants and animals (Wei *et al.*, 1998; Houben *et al.*, 1999; Kaszas and Cande, 2000; Manzanero *et al.*, 2000; Paula *et al.*, 2013). In animals, this mark is essential for the condensation of meiotic chromosomes and their segregation, because experiments show that artificially reduced H3S10 phosphorylation levels lead to meiotic problems and sterile phenotypes (Wei *et al.*, 1999). In plants, the mitotic H3S10p chromatin mark is localized in (peri)centromeric regions of chromosomes, and in the first meiotic division the mark tends to occupy the whole chromosome (Manzanero *et al.*, 2000). More variable patterns have been reported in the second meiotic division, when histone phosphorylation levels are generally reduced compared with those in the first meiotic division, and differences are likely to occur in the function of H3S10p between animals and plants. The other phosphorylated amino acids of histone H3 linked with nuclear division are the serine at position 28 (H3S28p) and threonine at position 3 (H3T3p) (Houben *et al.*, 2007; Sawicka and Seiser, 2012). As demonstrated in *Secale cereale* (rye), the H3S10p and H3S28p sites frequently co-localize and are likely to influence sister chromatid cohesion (Manzanero *et al.*, 2000; Gernand *et al.*, 2003). Interestingly, at meiosis II, no phosphorylation of H3S10 and H3S28 occurs on chromatids derived from prematurely separated univalents (Manzanero *et al.*, 2000). Although notable exceptions to this trend exist (Fernandes *et al.*, 2008), it is likely that loss of phosphorylation is needed for proper chromatid pair segregation.

*Rosa canina* L. and *Rosa rubiginosa* L. (2*n* = 5*x* = 35) represent typical examples of dogroses performing asymmetrical meiosis. The species belong to different subsections [*Caninae* and *Rubigineae* (DC.) Christ, respectively], whose positions on several phylogenies imply that they arose by independent hybridization events of regularly sexual progenitor species, and thus, canina meiosis arose at least twice independently (Wissemann and Ritz, 2005; Bruneau *et al.*, 2007; Fougere-Danezan *et al.*, 2015). Cytogenetic studies using ribosomal and satellite DNA markers corroborated this hypothesis, because chromosomes form bivalents in *Caninae* and univalents in *Rubigineae* and vice versa (Herklotz *et al.*, 2018; Vozárová *et al.*, 2021; Lunerová *et al.*, 2020).

Detailed studies of asymmetrical meiosis in dogroses are scarce, and virtually no reports exist on epigenetic modification of their chromosomes. To address this lack of studies, we analysed meiotic and mitotic chromosomes of *R. canina* and *R. rubiginosa* by immunostaining with antibodies recognizing modified residues of histone H3. In addition, transmission of chromosomes during meiosis was analysed by rDNA markers. We found that univalent and bivalent chromosomes acquire differential phosphorylation statuses in the first meiotic division, underlining the role of epigenetics in their differential segregation.

## MATERIALS AND METHODS

### Plant material

Fresh flower buds from wild rose bushes of *R. canina* (subsect. *Caninae*) were sampled from three individuals of one population in Brno, Czech Republic (CZ; WGS84: 49.2310°N, 16.5957°E), and a single individual from a population around Gatersleben, Germany (DE; 51.827342°N, 11.275076°E). Buds of *R. rubiginosa* (subsect. *Rubigineae*) were isolated from an individual originally from Balsgaard, Sweden, which has been cultivated further in the garden of the institute (IBP, Brno, CZ; 49.2207°N, 16.5809°E), and *Rosa nitida* Willd. from a commercial source was grown in the garden of the institute (IBP, Brno, CZ; 49.2207°N, 16.5809°E). All plants were grown outside, in natural conditions. Voucher specimens for Czech *R. canina* (GLM-P-0181117) and *R. rubiginosa* (GLM-P-0181118) were deposited in the Herbarium Senckenbergianum Goerlitz (GLM). The anthers from flower buds ~0.5 cm in length were harvested in May/June of 2019 and 2023 and used for downstream experiments. Approximately 300 flower buds of *R. canina*, 50 of *R. rubiginosa* and 15 of *R. nitida* were screened per year. Buds were cut, and anthers were checked for meiotic activity by fluorescence microscopy after 4ʹ,6-diamidino-2-phenylindole (DAPI) staining (H-1200, Vectashield with DAPI; Vector Laboratories, UK). Only slides with a sufficient number of meiocytes were selected for further analyses. To observe histone H3 phosphorylation in somatic tissue, additional material from root tips of *R. canina* and *R. nitida* was collected from young plants. As examples of a species with standard meiosis, we analysed two diploid (both 2*n* = 2*x* = 14) representatives from section *Rosa*, i.e. *R. rugosa* Thunb. (pollen fertility) and *R. nitida* (immunohistochemistry) (both grown at IBP, Brno, CZ; 49.2212153°N, 16.5794517°E).

### *Fluorescence* in situ *hybridization*

Preparations of male meiocytes were obtained from anthers of *R. canina* and *R. rubiginosa* following a protocol described by Lunerová and Vozárová (2023). Fresh flower buds were fixed in a solution of ethanol and acetic acid (3:1) and stored in 70 % ethanol at −20 °C. Anthers were washed with 1 % (w/v) polyvinylpyrrolidone 40 (PVP-40; Sigma–Aldrich Chemie GmbH, Taufkirchen, Germany) and 0.5 % (v/v) Triton X-100 for 15–20 min, followed by enzymic digestion overnight at 4 °C in 1 % (w/v) cellulase Onuzuka R-10 (Serva GmbH, Heidelberg, Germany), 0.2 % (w/v), pectolyase Y-23 (Sigma‒Aldrich Chemie GmbH, Taufkirchen), 0.5 % (w/v), hemi-cellulase (Sigma‒Aldrich) and 0.5 % (w/v) macerozyme R-10 (Duchefa Biochimie, Haarlem, The Netherlands) dissolved in citric buffer (0.04 m citric acid and 0.06 m sodium citrate).

The fluorescence *in situ* hybridization (FISH) procedures followed protocols described by Lim *et al.* (2005) and Herklotz *et al.* (2018). Briefly, anthers were macerated on a slide, squashed in a drop of 70 % acetic acid and fixed by snap freezing in liquid nitrogen. A cloned 1.7 kb fragment of the *Solanum lycopersicum* 18S rRNA gene (GenBank X51576.1) was used as a probe. The probe was directly labelled by nick translation using Atto647N (Jena Bioscience, Jena, Germany; pseudo-coloured to red in the figures). The hybridization mixture contained 100 ng labelled probe, 50 % (v/v) formamide, 10 % (w/v) dextran sulphate and 2× saline–sodium citrate (SSC) buffer. Chromosomes were counterstained with DAPI and hybridized against the probe overnight at 37 °C. The post-hybridization washing steps were carried out in a water bath (42 °C) in the dark. The slides were washed twice in 2× SSC for 5 min (stringency ~60 %), followed by a more stringent wash in 0.1× SSC (stringency ~82 %) twice for 5 min. The slides were then removed from the water bath (still in a dark box), cooled to room temperature and washed in 2× SSC for 5 min, followed by 4× SSC with 0.1 % Tween 20 for 7 min and a final brief wash in 1× PBS.

All slides were examined under an Olympus Provis AX70 epifluorescence microscope (Metasystems, Germany). The imaging software used was ISIS (MetaSystems), and images were optimized for contrast and brightness with Adobe Photoshop PS2021.

### Immunostaining procedures

Histone modification marks were immunolocalized according to several combined protocols (Houben *et al.*, 2003; Demidov *et al.*, 2014). Anthers from young buds were dissected and fixed for 20–25 min in freshly prepared 4 % (w/v) paraformaldehyde with 0.1 % (w/v) Triton X-100 in 1× phosphate-buffered saline (1× PBS, pH 6), followed by additional incubation on ice for 20 min, if necessary. The anthers were washed three times for 15 min in an ice-cold mixture containing 2 % (w/v) PVP-40 and 0.5 % (v/v) Triton X-100 diluted in 1× PBS and three times for 5 min in ice-cold 1× PBS. Tissues were then digested with a mixture of enzymes containing 2.5 % (w/v) hemi-cellulase (Sigma-Aldrich), 2.5 % (w/v) cellulase Onozuka R-10 (Serva), 2.5 % (w/v) macerozyme (Duchefa) and 1.0 % (w/v) pectolyase Y-23 (Sigma‒Aldrich) dissolved in 1× PBS or 1× microtubule stabilizing buffer (for microtubule detection) overnight at 4 °C. Enzymes were then removed, and tissue was disrupted by gentle tapping with a metal rod to generate a suspension of cells. The mixture was filtered through a 30 µm mesh filter. To reduce polyphenolic contaminants of microscopic preparations, the anther mash was incubated in an ice-cold solution of 2 % PVP-40 and 0.5 % Triton X-100 in 1× PBS for two rounds of 5 min each in a rotating shaker, followed by washes with 1× PBS for three rounds of 5 min each. For microtubule detection, we used 1× microtubule stabilizing buffer as an incubation buffer. After washing steps, the meiocyte suspension was placed on poly-l-lysine-coated slides (Sigma‒Aldrich) and squashed between a glass slide and coverslip. After freezing in liquid nitrogen, the coverslip was removed, and slides were transferred immediately into 1× PBS. For immunostaining, the slides were incubated in blocking buffer [1× PBS with 5 % (w/v) bovine serum albumin and 0.1 % (v/v) Triton X-100] in a prewarmed humid chamber (for immunochemistry) at 37 °C for 60 min. Primary antibodies were added to slides and incubated in a humid chamber at 4 °C overnight, followed by subsequent washes (three times for 5 min in 1× PBS with 0.1 % Tween 20 on ice). The slides were incubated with secondary antibodies for 2 h at 37 °C. Slides were washed three times in 1× PBS for 5 min each and subsequently dehydrated in an ethanol series (70, 90 and 100 %). The slides were air dried in a dark box, mounted in Vectashield containing DAPI and observed under a microscope. All primary and secondary antibodies were diluted in 1× PBS, 1 % bovine serum albumin and 0.1 % Tween 20. Working dilutions of primary antibodies are listed in Table 1. The secondary antibodies included anti-rabbit IgG Cy3 conjugate (Jackson Immunoresearch, USA; catalogue number 111167003) and anti-rabbit IgG fluorescein isothiocyanate (FITC) conjugate (Jackson Immunoresearch, USA; catalogue number 111097003), both diluted 1:300. For simultaneous immunostaining, we used a combination of anti-mouse IgG FITC conjugate (Sigma‒Aldrich; catalogue number F0257; 1:200 dilution) and anti-rat IgG Cy3 conjugate (Thermo Fisher Scientific, Invitrogen, USA; catalogue number A10522; 1:1000 dilution) antibodies.

**Table 1.** List of antibodies and their targets used in cytogenetic analyses.

Primary antibody	Working dilution	Origin	Source/product number
α-tubulin	1:500	Rat	Invitrogen/MA1-80017^a^
H3S10p	1:500	Rabbit	ActiveMotif/39253^b^
H3S28p	1:800	Rat	ActiveMotif/39148^b^
H3T3p	1:400	Rabbit	Millipore/07-424^c^
H3K27me3	1:200	Mouse	Abclonal/A16199^d^
H3K4me3	1:200	Rabbit	Abcam/ab8580^e^

Suppliers:

^a^Thermofisher Scientific, Invitrogen, Waltham, MA, USA;

^b^ActiveMotif, Waterloo, Belgium;

^c^Merck KGaA, Millipore, USA;

^d^Abclonal Technology, Woburn, MA, USA;

^e^Abcam plc, Cambridge, UK.

The intensity signals of immunostaining were quantified in TIFF images using ImageJ software (Rasband, 1997-2018). The numerical pixel values of fluorescence intensity were obtained from manually annotated chromosomes after selection for DAPI (blue), FITC (green) and Cy3 (red) fluorescence filters. The mean grey value was calculated as the sum of the grey values of all the pixels in the selection divided by the total number of pixels. Box plots were constructed in RStudio (BoxPlotR, 2013; R Development Core Team, 2013) Mann‒Whitney non-parametric tests were performed using an online calculator (StatisticsKingdom, 2017). A total of 639 meiocytes from several *R. canina* individuals and one *R. rubiginosa* individual were examined with the antibodies listed in Table 1. A summary of the samples and analyses is given in Supplementary Data Table S1.

### Viability staining of pollen grains

For pollen vitality tests, mature pollen grains were collected from fresh yellow anthers of closed flowers, air dried for 5 days and stored in reaction tubes until use. To estimate the vitality of pollen grains, we followed the protocol of Alexander (1969). Briefly, the staining reagent (1 mL) contained 10 µL of Malachite Green, 50 µL of Acid Fuchsin and 5 µL of Orange G diluted in 95 µL of absolute ethanol (Sigma‒Aldrich Chemie GmbH, Taufkirchen, Germany), 250 µL of glycerol, 40 µL of acetic acid and 550 µL of distilled water. Pollen grains were incubated with the reagent in rotating collection tubes for 1 h and counted in a Burker chamber.

## RESULTS

### The course of canina meiosis as analysed by FISH

**Fig. 2.**
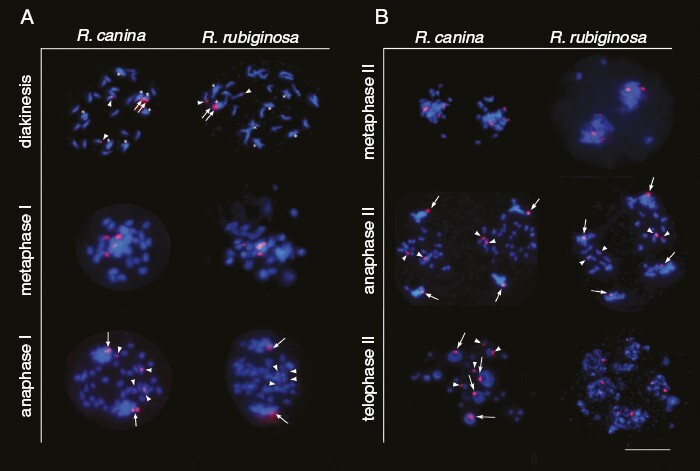
Fluorescence *in situ* hybridization of *Rosa canina* and *Rosa rubiginosa* meiotic chromosomes. Sites of 18S rDNA probe hybridization are shown in red. Arrows and arrowheads mark positions of rDNA loci on bivalent and univalent chromosomes, respectively. (A) First meiotic division. (B) Second meiotic division. In diakinesis, bivalent chromosomes are marked by asterisks. In metaphase, individual chromosome types could not be resolved. Note the separation of univalent-derived chromatids in anaphase I (equatorial position). Note that only a few univalents were retained in *R. rubiginosa* anaphase II. Note the newly formed nucleoli surrounded by rDNA (arrows) in telophase II; micronuclei containing univalent loci (arrowheads) lacked nucleoli. Scale bar: 10 µm.

Fluorescence *in situ* hybridization in combination with DAPI staining of chromatin was used to identify chromosomes at different stages of meiosis in *R. canina* and *R. rubiginosa*. In diakinesis, all seven homologous chromosome pairs (forming bivalents) resembled thick rods along their entire lengths (Fig. 2A, top preparations, asterisks). In contrast, most of the 21 unpaired chromosomes (univalents) tended to form thin fibres exhibiting relatively weak staining. The 18S rDNA probe hybridized to four sites, two on bivalents and two on univalents. No multivalents were detected in diakinesis. All four 18S rDNA sites were also visible in metaphase I (Fig. 2A, middle panel). In anaphase I, homologous pairs in bivalents separated by moving to the poles as in regular meiosis. In contrast, 21 univalents were separated into 42 chromatids that had delayed migration (Fig. 2A, bottom). A single (corresponding to tightly linked homologues) or a double (corresponding to loosely linked homologues) rDNA signal was visualized in each bivalent sector. In the univalent sector, there were four rDNA signals (arrowheads) derived from a split of chromatid pairs of the two nucleolus organizer regions (NOR)-positive chromosomes. We did not observe cytokinesis between meiosis I and meiosis II. In metaphase II, each of the two daughter nuclei inherited four 18S rDNA sites (two on bivalents and two on univalents) (Fig. 2B, top). In anaphase II, the bivalents separated into chromatids (arrows) and migrated to the poles (Fig. 2B, middle). In contrast, the univalent-derived chromatids (arrowheads) remained between the two meiotic spindles and probably never reached the poles. There were eight 18S rDNA signals indicating separation of chromatids of all NOR chromosomes at this stage. Additionally, at telophase II (Fig. 2B, bottom), eight rDNA signals were visible. The four signals were located in large nuclei with nucleoli probably representing premature microspores, and another four signals were visible in micronuclei. In contrast to Klášterská (1971), we did not observe nucleolus-like bodies in the cytoplasm, and all rDNA loci seemed to associate with chromosomes. Among the univalent chromosomes, one rDNA locus was consistently missing, indicating that one of the subgenomes lacked essential rRNA genes and was no longer able to provide fertile gametes. In other *R. canina* accessions, all five rDNAs are still present (Lim *et al.*, 2005; Herklotz *et al.*, 2018), although one of them is extremely small, in line with the loss of rDNA copies observed in many allopolyploids (Garcia *et al.*, 2017). In summary, premature separation of univalent chromatid pairs in phase I has been confirmed by rDNA markers in two dogrose lineages of independent origin.

Finally, we determined the pollen viability of 5*x R. canina*, 5*x R. rubiginosa* and 2*x R. rugosa* (Supplementary Data Table S2). Pollen was collected from the same population over four consecutive years (2020–2023). The pollen viability of the diploid *R. rugosa* was high (80–90 %) and relatively stable over the years. In contrast, the viability of pentaploid dogrose pollen ranged from 17 to 50 % in *R. canina* and from 30 to 45 % in *R. rubiginosa*.

### Histone H3 phosphorylation marks in diakinesis/early metaphase I in canina and non-canina meiosis

**Fig. 3.**
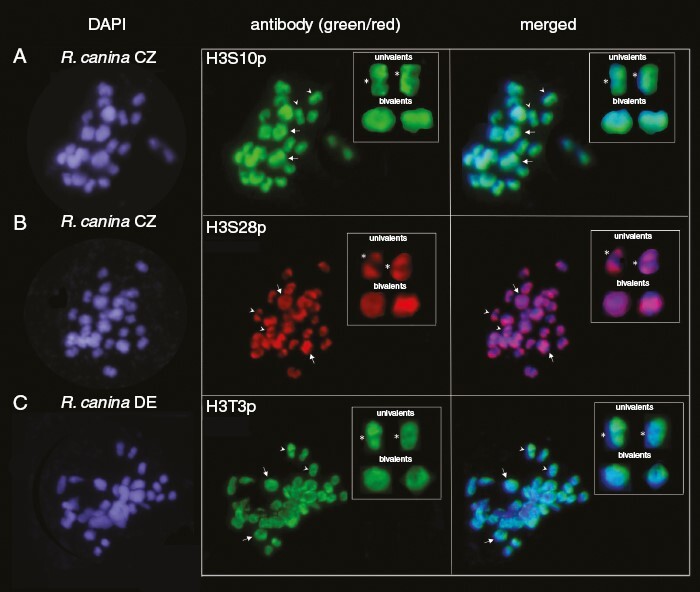
Immunolocalization of H3 histones phosphorylated at serine 10 (H3S10p, green), serine 28 (H3S28p, red) and threonine 3 (H3T3p, green) residues. The chromosomes correspond to late diakinesis/metaphase I of *Rosa canina* meiosis. The bivalent pairs and univalents are marked with arrows and arrowheads, respectively. Rectangular insets: selected univalent and bivalent chromosomes showing a typical immunostaining pattern. Asterisks in the middle panel indicate the positions of putative centromeres. Note the weak immunostaining of (peri)centromeric regions in univalent chromosomes. Scale bar: 10 µm.

Major changes in histone H3 by phosphorylation are expected to occur at the early stages of the first meiotic division (Fuchs *et al.*, 2006). Therefore, we analysed late diakinesis/early metaphase I nuclei by immunostaining of nuclei with antibodies against phosphorylated H3S10p, H3S28p and H3T3p histones. In *R. canina*, both bivalents and univalents showed positive staining with all three marks (Fig. 3). The bivalent chromosomes were intensely stained with the H3S10p and H3S28p antibodies along their whole lengths (Fig. 3A, B, arrows). In univalents (arrowheads), most phosphorylation signals were localized on the chromosome arms and in distal regions, while the proximal regions were less intensively stained, particularly with the H3S28p antibody (Fig. 3B). Selected chromosomes are enlarged in the insets in Fig. 3. Staining patterns were reproducible between independent meiotic cells of *R. canina* (Supplementary Data Fig. S1A, B).

**Fig. 4.**
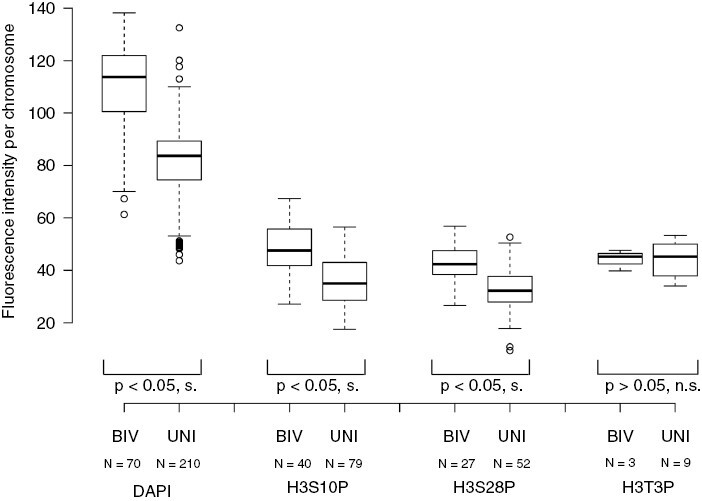
Statistical evaluation of fluorescence intensity after the staining of chromosomes at diakinesis with 4ʹ,6-diamidino-2-phenylindole (DAPI) (DNA) and antibodies against phosphorylated histone H3. The boxplot diagrams show mean fluorescence intensities calculated for the bivalent (BIV) and univalent (UNI) chromosomes. The data are taken from Supplementary Data Table S1. Lines in boxes correspond to medians. The top and bottom ends of the box indicate the top and bottom quartiles, respectively, whereas the upper and lower whiskers indicate the smallest and largest values of the set. ‘*N*’ is the number of counted chromosomes. The abbreviations ‘s.’ and ‘n.s.’ indicate significant and non-significant differences, respectively, at the >95 % probability level (Mann‒Whitney *U* test).

To quantify fluorescence signals, we evaluated the fluorescence intensity of DAPI (DNA) and immunostaining signals across the chromosomes (Fig. 4). It was evident that the bivalent chromosomes showed stronger fluorescence (calculated as an average signal per area) than the univalents (Mann‒Whitney *U* test, two-tailed: for DAPI, *N* = 280, *z*-value = −9.76, *P* = 0.00010; for H3S10p, *N* = 119, *z*-value = −5.6; *P* = 6.979 × 10^−9^; and for H3S28p, *N* = 79, *z*-value = −4.43, *P* = 0.00028). Similar results were obtained for *R. rubiginosa* diakinesis chromosomes stained with the anti-H3S10p antibody, as shown in Supplementary Data Fig. S2. Staining of chromosomes was also obtained with the H3T3p antibody (Fig. 3C). However, the signals were more diffuse in appearance, and in comparison to H3S10p and H3S28p, the differences between bivalents and univalents were non-significant (Mann‒Whitney *U* test, two-tailed: *N* = 12, *z*-value = 0.17, *P* = 0.8636).

In the diploid *R. nitida* (2*n* = 2*x* = 14), a species with standard meiosis, all 14 chromosomes formed bivalents that were heavily stained with the H3S10p antibody across their whole lengths in metaphase I (Supplementary Data Fig. S3A).

### *Immunolocalization of α-tubulin and H3S10p at different meiotic stages in the* R. canina *and* R. rubiginosa *pentaploids*

**Fig. 5.**
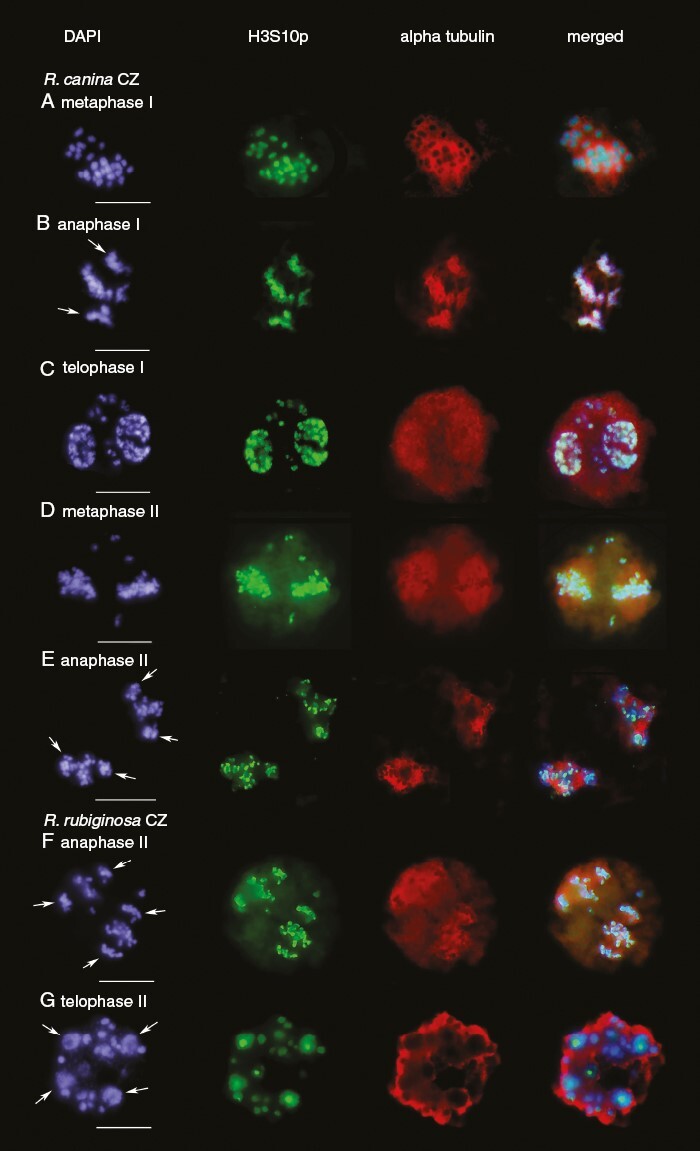
Immunolocalization of histone H3S10p marks (green) and α-tubulin (red) on chromosomes during the first and second meiotic divisions. (A–E) *Rosa canina*. (F, G) *Rosa rubiginosa*. Note the association of α-tubulin with both bivalent (leading) and univalent (lagging) chromosomes in anaphase I (B) and to a much lesser extent in anaphase II (E, F). Note the relatively strong H3S10p signals in metaphase chromosomes (A, D). The bivalents, bivalent-derived chromatids and major tetrad cells are depicted by arrows. Scale bars: 10 µm.

To visualize the relative position of microtubules and chromosomes during canina meiosis, we used immunostaining with antibodies against α-tubulin and H3S10p. Therefore, a more complete set of meiotic stages is shown for *R. canina* (Fig. 5A–E); meiotic stage II is also shown for *R. rubiginosa* (Fig. 5F, G). The polar view of *R. canina* metaphase I chromosomes showed an overlap of H3S10p signals with the α-tubulin signals (Fig. 5A). Likewise, in anaphase I, both bivalent and lagging univalents were associated with α-tubulin (Fig. 5B). In the second meiotic division, the H3S10p signals were clearly visible up to the anaphase II stage (Fig. 5D–F). In contrast to that in anaphase I, the association of chromatids with α-tubulin was not as pronounced in anaphase II, particularly for univalents that remained at the equator (Fig. 5E, F). At telophase II, we observed diffuse H3S10p signals of decondensing chromosomes (Fig. 5G; Supplementary Data Fig. S4A). Fluorescence signals became almost undetectable in polyads (Supplementary Data Fig. S4B).

### Histone H3 phosphorylation patterns in somatic chromosomes

To analyse the histone H3 phosphorylation profiles in somatic tissues, we analysed root tip nuclei from *R. canina* pentaploid and *R. nitida* diploid plants. The metaphase chromosomes were immunostained with antibodies against H3S10p, H3S28p and H3T3p. In both *R. canina* (Supplementary Data Fig. S5A, B) and *R. nitida* (Supplementary Data Fig. S3B), the H3S10p and H3S28p signals were localized preferentially to pericentromeric positions. The H3T3p phosphorylation signals were more dispersed (Supplementary Data Fig. S5C) and appeared to be somewhat stronger at (peri)centromeric regions.

### Euchromatic and heterochromatic histone marks on dogrose chromosomes

Next, we wondered whether bivalent and univalent chromosomes differ in euchromatin/heterochromatin landscapes. Therefore, we examined euchromatic H3K4me3 and heterochromatic (facultative) H3K27me3 marks on metaphase I chromosomes (Supplementary Data Fig. S6). In both *R. canina* (Supplementary Data Fig. S6A) and *R. rubiginosa* (Supplementary Data Fig. S6B), the H3K4me3 signals were located mostly at interstitial and distal positions, often on both arms. This pattern corresponded to that of H3S28p staining (for comparison, see Fig. 3B). Immunostaining of the H3K27me3 mark produced diffuse patterns with several spot-like signals in distal regions (Supplementary Data Fig. S6C, D). Staining of somatic root tip nuclei with antibodies against the H3K4me3 and H3K27me3 marks resulted mostly in whole chromosome patterns with some local maxima and minima (Supplementary Data Fig. S7). In general, there was no or difference only a small difference in eu- and heterochromatin immunostaining patterns between mitotic and meiotic cells and between univalent and bivalent chromosomes, at least for the two histone H3 marks investigated.

## DISCUSSION

In asymmetrical canina meiosis, undivided univalents are transmitted to offspring via the female gamete, but they are eliminated during microsporogenesis and do not occur in mature pollen. The mechanism of univalent chromosome elimination in pollen mother cells has often been overlooked. On the basis of molecular cytogenetics methods, this study provides some insights into the mechanisms of univalent chromosome behaviour during male canina meiosis.

### Molecular cytogenetics reveals equational division of univalents in the first meiotic division, followed by their gradual elimination in the second meiotic division

We investigated individual stages of canina meiosis in *R. canina* and *R. rubiginosa* (both 2*n* = 5*x* = 35). The rDNA FISH marker clearly revealed separation of univalent chromatids in anaphase I (Fig. 2A, bottom). The chromatids migrated as laggers, corroborating the findings of all classical cytological studies (Blackburn and Harrison, 1921; Täckholm, 1922; Klášterská and Natarajan, 1974*a*; Roberts, 1975). We were unable to detect non-disjunction of any univalent chromosome, indicating that each daughter nucleus receives seven bivalent-derived chromatids and 21 univalent-derived chromatids. Thus, the first meiotic division of canina meiosis includes the reductional division of homologous bivalent chromosomes and equational division of univalent chromosomes. In contrast to *Oenothera* (Onagraceae), in which some genotypes also exhibit irregular meiosis due to permanent reciprocal translocations (Golczyk *et al.*, 2008), dogroses do not appear to have multivalents in diakinesis, with their occurrence seemingly limited to the earlier prophase stage (Supplementary Data Fig. S8). As in other allopolyploids, these structures might be fixed later or removed by selection (Cifuentes *et al.*, 2010; Soares *et al.*, 2021). In the second meiotic division, former univalents exist in the form of chromatids that cannot be divided further. However, they are still capable of binding microtubules migrating irregularly across the meiotic plate. In anaphase II, we observed irregular counts of prematurely separated chromatids, chromatids located outside the meiotic plate and an uneven distribution of rDNA sites (Figs 2B and 4E, F). These features suggest a near-to-stochastic distribution of univalent chromosomes in meiosis II. We noted variable (~21 ± 5) distributions of chromatids between daughter cells in anaphase II. It is possible that cells with higher chromosome counts falsely lead to the inference of univalent replication between meiosis I and II (Täckholm, 1922). Thus, univalents are eliminated gradually at the terminal stage of male meiosis, probably owing to a cascade of events triggered in its early stages.

### Bivalent and univalent chromosomes contain both active and inactive epigenetic marks

In contrast to histone phosphorylation marks, methylation marks have been studied less often in meiotic cells. In our experiments, the H3K4me3 euchromatin marker was abundantly present on chromosomes at diakinesis and early metaphase I (Supplementary Data Fig. S6). Zygotene/pachytene chromosomes were also strongly stained with antibodies against di- and trimethylated lysine in tobacco (Mursalimov *et al.*, 2019). These results support the notion that active H3K4me3 marks are not erased during meiotic division and might even be enhanced to some extent. In this context, H3K4me3 chromatin might be associated with meiotic recombination sites, as shown for *Saccharomyces cerevisiae* (Borde *et al.*, 2009). It remains to be determined whether the observed hypermethylation of H3K4 is correlated with histone acetylation, which has been shown to be enhanced in prophase in some systems (Feitoza *et al.*, 2017), and whether it reflects the transcriptional activity of genes residing in univalent chromosomes.

### Reduced histone H3 phosphorylation of univalent chromatin in meiotic phase I

Histone H3 phosphorylation at S10 and S28 in plants is usually found at (peri)centromeric regions, where it functions in mitotic and meiotic sister chromatid cohesion and successful chromosome segregation (Manzanero *et al.*, 2000; Rossetto *et al.*, 2012; Loginova and Silkova, 2017). Strikingly, in the first meiotic division, univalent chromosomes showed only weak phosphorylation levels in these regions (Figs 3 and 4), and most signals were localized to the interstitial and distal parts of chromosomes. This pattern contrasts with that of the bivalents, which showed strong histone phosphorylation signals spreading from (peri)centromeric regions across the entire chromosome length.

How can the unusual staining patterns of univalent chromosomes be explained? Given that phosphorylation is a hallmark of functional (peri)centromeres, it is possible that their centromeres are impaired or degenerated. However, the following observations argue against this hypothesis. First, in root mitotic cells, all chromosomes showed the canonical (peri)centromeric localization of the H3S10p phosphorylation mark (Supplementary Data Fig. S5). Furthermore, there was no evidence for abnormal mitosis (Lim *et al.*, 2005; Herklotz *et al.*, 2018). Second, the (peri)centromeric CANR4 repeat previously found in most *Caninae* genomes occurs in both univalent and bivalent chromosomes and is more abundant in the former (Lunerová *et al.*, 2020). Hence, the univalent chromosomes seem to harbour functional centromeres, and their abnormal phosphorylation patterns seem to be linked to the first meiotic stage. It will be interesting to analyse H3 phosphorylation in prophase to see whether aberrant phosphorylation is a cause or consequence of pairing failure.

Intriguingly, the reduction in histone H3S10 and H3S28 phosphorylation in (peri)centromeric regions preceded a split of univalent chromatid pairs in the first meiotic division. Centromeres are chromosomal regions that interact with the spindle apparatus during each nuclear division to ensure the disjunction of chromosomes (Talbert and Henikoff, 2020). It has been shown that the reduction in histone H3S10 phosphorylation in these regions is associated with chromosome defects and meiotic abnormalities in animals (Wei *et al.*, 1999). Thus, one of the functions of histone phosphorylation could be to assist sister chromatid cohesion, perhaps through increased chromatin condensation. In comparison to univalents, bivalents were strongly stained with DAPI in diakinesis (Fig. 4) and prophase (Supplementary Data Fig. S8). This finding is consistent with some earlier cytological observations. For example, Erlanson (1933) attributed non-synapsis of univalent chromosomes to their late condensation at prophase I. Later, irregular contraction of chromosomes in the first meiotic division was reported in several *Canina* species (Klášterská and Natarajan, 1974*a*, *b*). It should be mentioned that full decondensation of univalents has never been observed in meiosis, and heterochromatic regions (satellites and rDNA) seem to be condensed normally (Fig. 2; Lunerová *et al.*, 2020).

It might be informative to compare the behaviour of canina chromosomes with that in other systems bearing non-pairing chromosomes in their karyotypes, such as B chromosomes. These supernumerary chromosomes occur in both plants and animals, exhibiting a non-Mendelian mode of transmission, and they do not recombine with autosomes. There are notable differences between both systems. For example, the canina univalents divide equationally in phase I, whereas the B chromosomes, in general, undergo non-disjunction and migrate as dyads to one of the poles (Chiavarino *et al.*, 2000; Chen *et al.*, 2022). Interestingly, canina histone H3S10 phosphorylation is not completely erased in the second meiotic division (Fig. 5E, F), indicating that separation of chromatid pairs does not lead to complete loss of H3S10p chromatin marks. This contrasts with the situation in maize and rye (Manzareno *et al.*, 2000), where the B chromosomes lack H3S10 phosphorylation completely. The immunostaining patterns of canina meiotic chromosomes somewhat resemble those of the grasshopper *Eyprepocnemis plorans*, whose univalent X-chromosome retains H3 phosphorylation throughout the meiotic cycle. Of note, similar to *R. canina*, *E. plorans* exhibits equationally dividing univalents at the first division (Rebollo and Arana, 1995).

### A hypothetical model of chromosome behaviour in canina male meiosis

**Fig. 6.**
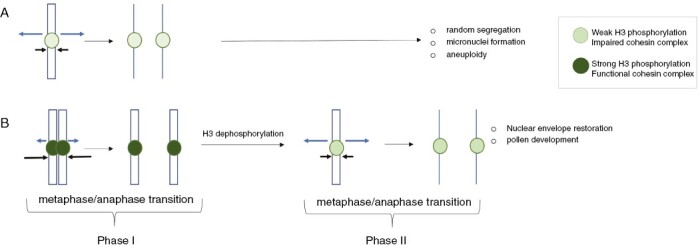
A hypothetical model showing the fate of univalents (A) and bivalents (B) in canina meiosis. A role of histone H3 phosphorylation (S10, S28) in chromatid separation is proposed during the metaphase/anaphase transitions. Dark green circles indicate highly phosphorylated (peri)centromeric chromatin. Light green circles indicate no or weakly phosphorylated (peri)centromeric chromatin. Black arrows indicate cohesive force maintaining the integrity of chromatids. Blue arrows indicate repulsive force of the kinetochore.

It is well established that the cohesin complex plays a central role in the successful transmission of chromosomes through meiosis (Bolanos-Villegas, 2021). The complex includes meiosis-specific subunits, such as the meiotic α-kleisin REC8/SYN1 (Castellano-Pozo *et al.*, 2020), which are deposited on chromatin during leptotene. The synaptonemal complex (SC) is then assembled fully between paired homologous chromosomes at pachytene. Centromeric cohesion is protected by the conserved SUGOSHIN (SGO1/2 in *Arabidopsis*) family of proteins until anaphase II (Cromer *et al.*, 2013). Based on these suppositions, a hypothetical model of chromosome behaviour in canina meiosis is proposed in Fig. 6. In anaphase, there is a balance between opposing forces, a mitotic spindle on the one hand and cohesins holding sister centromeres on the other. However, the loading of cohesins onto the chromosomes could be reduced or even fail in dogrose univalents, which are structurally heterogeneous and lack homologous partners (V. Heklotz (Senckenberg Museum of Natural History, Senckenberg, Germany), J. Lunerová (Czech Academy of Sciences, Czechia), A. Marquez (MPI, Germany), C.M. Ritz (Senckenberg Museum of Natural History, Senckenberg, Germany), unpubl. res.). Thus, in univalents, a repulsion force of the mitotic spindle dominates over the cohesion force, perhaps as a result of aberrant chromatin modifications and cohesin insufficiencies. This apparently leads to their equation division in anaphase I. In contrast, the cohesive force associated with hyperphosphorylated bivalent chromatin keeps their chromatids together. Certainly, in the second meiotic division, bivalent chromosomes gradually lose phosphorylation, degrade residual cohesins and, as in other systems, divide equationally.

### Conclusions

We found differences in the epigenetic histone H3 phosphorylation patterns between pairing and non-pairing chromosomes during canina asymmetrical meiosis. We hypothesize that reduced H3 phosphorylation levels of univalent chromosomes, particularly in pericentromeric regions, could destabilize the cohesion of chromatid pairs, leading to their premature separation in the first meiotic division.

## SUPPLEMENTARY DATA

Supplementary data are available at *Annals of Botany* online and consist of the following.

Figure S1: *Rosa canina* diakinesis/early metaphase I immunoanalysis with additional nuclei using antibodies against H3S10 (A) and H3S28 phosphorylation (B). Figure S2: *Rosa rubiginosa* early metaphase I immunostained with an antibody against H3S10p (A). Figure S3: immunolocalization of H3S10p marks in *Rosa nitida* (2*n* = 2*x* = 14), a species with standard meiosis. Figure S4: *Rosa canina* telophase II (A) and polyads (B) stained with antibodies against H3S10p (green) and anti-α-tubulin (red). Figure S5: analysis of histone H3 phosphorylation patterns in *R. canina* root tip nuclei. Figure S6: immunostaining of methylated histone H3 marks on meiotic chromosomes. Figure S7: immunostaining of *R. canina* root tip pro-/metaphase nuclei with antibodies against H3K4m3 (red) and H3K27m3 (green) histones. Figure S8: (A) *Rosa canina* prophase I chromosomes stained with 4ʹ,6-diamidino-2-phenylindole (DAPI). Table S1: summary of cytogenetic analyses performed in this study and basic statistics. Table S2: pollen fertility counts in pentaploid and diploid *Rosa* species.

mcad198_suppl_Supplementary_Tables_S1

mcad198_suppl_Supplementary_Tables_S2

mcad198_suppl_Supplementary_Figures_S1

mcad198_suppl_Supplementary_Figures_S2-S3

mcad198_suppl_Supplementary_Figures_S3

mcad198_suppl_Supplementary_Figures_S4

mcad198_suppl_Supplementary_Figures_S5-S6

mcad198_suppl_Supplementary_Figures_S6

mcad198_suppl_Supplementary_Figures_S7-S8
