# Supplementary material for: Epigenetic histone H3 phosphorylation marks discriminate between univalent- and bivalent-forming chromosomes during canina asymmetrical meiosis
Source: Ann Bot. 2023 Dec 21;133(3):435–46. doi: 10.1093/aob/mcad198 (PMC11006542; doi:10.1093/aob/mcad198)
Supplement: mcad198_suppl_Supplementary_Tables_S1 [file mcad198_suppl_supplementary_tables_s1.pdf]

Supplementary Table S1. Summary of cytogenetic analysis performed in this study and basic statistics.

	R. canina	*R. rubiginosa*
**Sum of all meiocytes observed (2021-2023)**	986	295
Average number of meiocytes per slide	2-5	3-6
Sum of meiocytes analyzed by immunostaining	639[a,b]	231[c]
**No. of evaluated diakinesis/metaphase I meiocytes for fluorescence intensity box plots (2021 – 2023):**		
DAPI	10	10
H3S10p	5	NA
H3S28p	4	NA
H3T3p	1	NA
**Staining: combined H3S10p/H3S28p**		
Number of slides	37	6
Sum of evaluated meiocytes	179[b]	30
**Staining: H3K4me3**		
Number of slides	5	1
Sum of evaluated meiocytes	20	4
**Staining: combined H3K27me3/H3S10p**		
Number of slides	1	1
Sum of evaluated meiocytes	6	4
**Staining: combined H3K27me3/H3S28p**		
Number of slides	4	NA
Sum of evaluated meiocytes	16	NA
**Staining: combined H3S28pme3/H3K4me3**		
Number of slides	2	1
Sum of evaluated meiocytes	10	5
**Staining: H3T3p**		
Number of slides	35	7
Sum of evaluated meiocytes	150[b]	35
**Staining: H3S10p**		

Number of slides	76	40
Sum of evaluated meiocytes	201	139
**Staining: H3S28p**<br>Number of slides	 2	 NA
Sum of evaluated meiocytes	2	NA
**Staining: DAPI**		
Number of slides	3	3
Sum of evaluated meiocytes	10	10

Footnotes:

[a] The majority of samples were from the Czech population of *Rosa canina.*

[b] Samples were collected from both Czech and German populations.

[c] Samples obtained from a single population of *R. rubiginosa*.
