# Supplementary material for: Epigenetic histone H3 phosphorylation marks discriminate between univalent- and bivalent-forming chromosomes during canina asymmetrical meiosis
Source: Ann Bot. 2023 Dec 21;133(3):435–46. doi: 10.1093/aob/mcad198 (PMC11006542; doi:10.1093/aob/mcad198)
Supplement: mcad198_suppl_Supplementary_Tables_S2 [file mcad198_suppl_supplementary_tables_s2.docx]

**Supplementary Table S2**. Pollen fertility counts in pentaploid and diploid Rosa species.

| **Year of sampling** | **Species** | **Fertile grains (%)** | **Sterile grains (%)** | **No. of pollen grains evaluated** |
| --- | --- | --- | --- | --- |
| **2020** |  |  |  |  |
|  | *R. rugosa* (2x) | 85 | 15 | 790 |
|  | *R. canina* (5x) | 52 | 48 | 620 |
|  | *R. rubiginosa* (5x) | 46 | 54 | 550 |
| **2021** |  |  |  |  |
|  | *R. rugosa* (2x) | 79 | 21 | 452 |
|  | *R. canina* 1 (5x) | 28 | 72 | 333 |
|  | *R. canina* 2 (5x) | 18 | 82 | 398 |
|  | *R. canina* 3 (5x) | 28 | 72 | 717 |
|  | *R. canina* 4 (5x) | 43 | 57 | 644 |
|  | *R. rubiginosa* (5x) | 27 | 73 | 165 |
| **2022** |  |  |  |  |
|  | *R. rugosa* (2x) | 94 | 6 | 905 |
|  | *R. canina* 1 (5x) | 23 | 77 | 1500 |
|  | *R. canina* 2 (5x) | 22 | 78 | 1586 |
|  | *R. canina* 3 (5x) | 17 | 83 | 1163 |
|  | *R. canina* 4 (5x) | 46 | 54 | 747 |
|  | *R. rubiginosa* (5x) | 37 | 63 | 402 |
| **2023** |  |  |  |  |
|  | *R. rugosa* (2x) | 94 | 6 | 889 |
|  | *R. canina* 1 (5x) | 36 | 64 | 1459 |
|  | *R. canina* 2 (5x) | 40 | 60 | 506 |
|  | *R. canina* 3 (5x) | 56 | 44 | 375 |
|  | *R. canina* 4 (5x) | 69 | 31 | 302 |
|  | *R. rubiginosa* (5x) | 39 | 61 | 1193 |
