# Supplementary material for: Epigenetic histone H3 phosphorylation marks discriminate between univalent- and bivalent-forming chromosomes during canina asymmetrical meiosis
Source: Ann Bot. 2023 Dec 21;133(3):435–46. doi: 10.1093/aob/mcad198 (PMC11006542; doi:10.1093/aob/mcad198)
Supplement: mcad198_suppl_Supplementary_Figures_S1 [file mcad198_suppl_supplementary_figures_s1.pptx]

## Slide 1
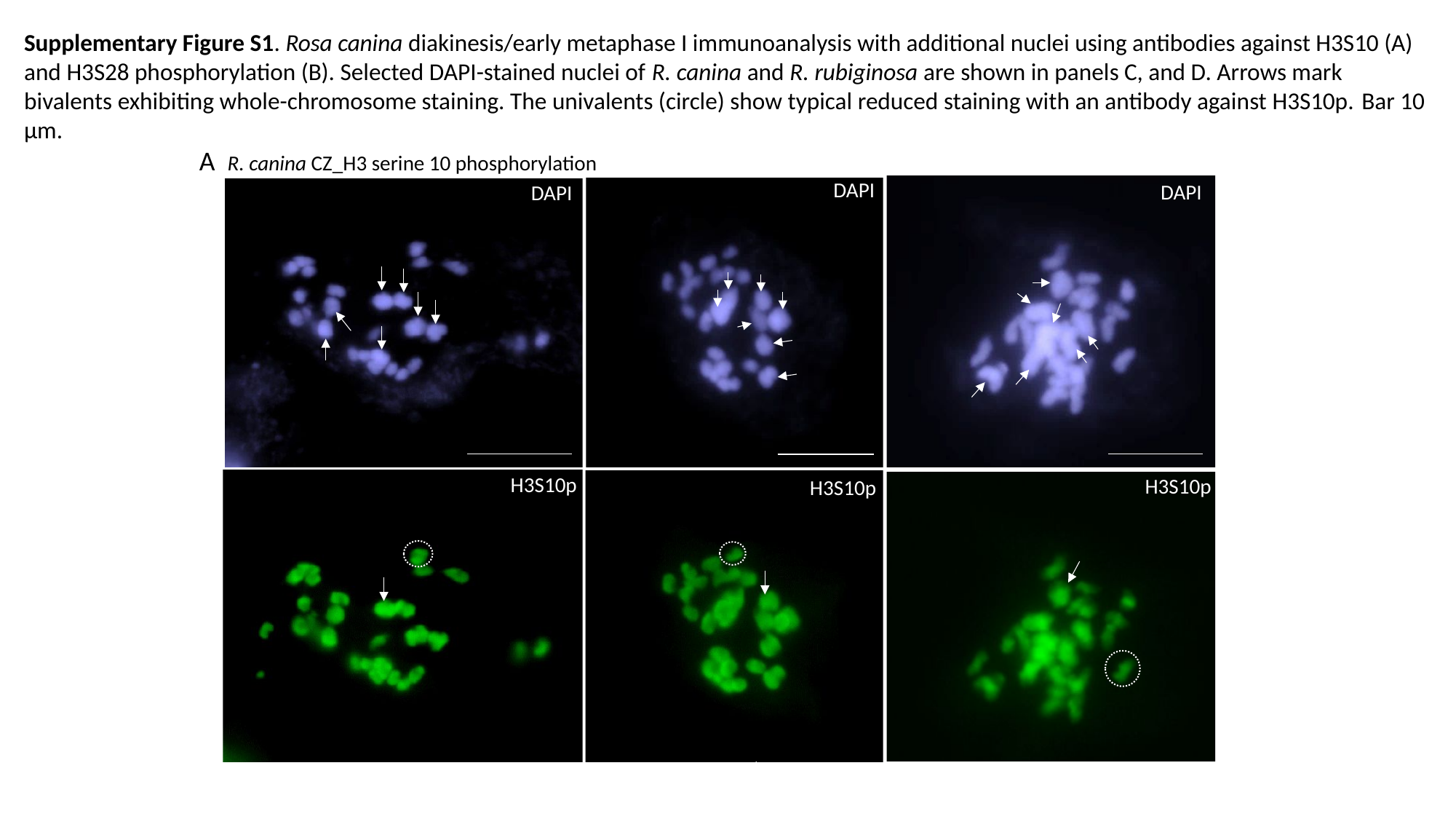

Supplementary Figure S1. Rosa canina diakinesis/early metaphase I immunoanalysis with additional nuclei using antibodies against H3S10 (A) and H3S28 phosphorylation (B). Selected DAPI-stained nuclei of R. canina and R. rubiginosa are shown in panels C, and D. Arrows mark bivalents exhibiting whole-chromosome staining. The univalents (circle) show typical reduced staining with an antibody against H3S10p. Bar 10 µm.
A R. canina CZ_H3 serine 10 phosphorylation
A
DAPI
DAPI
DAPI
DAPI
H3S10P
H3S10p
H3S10p
H3S10p

## Slide 2
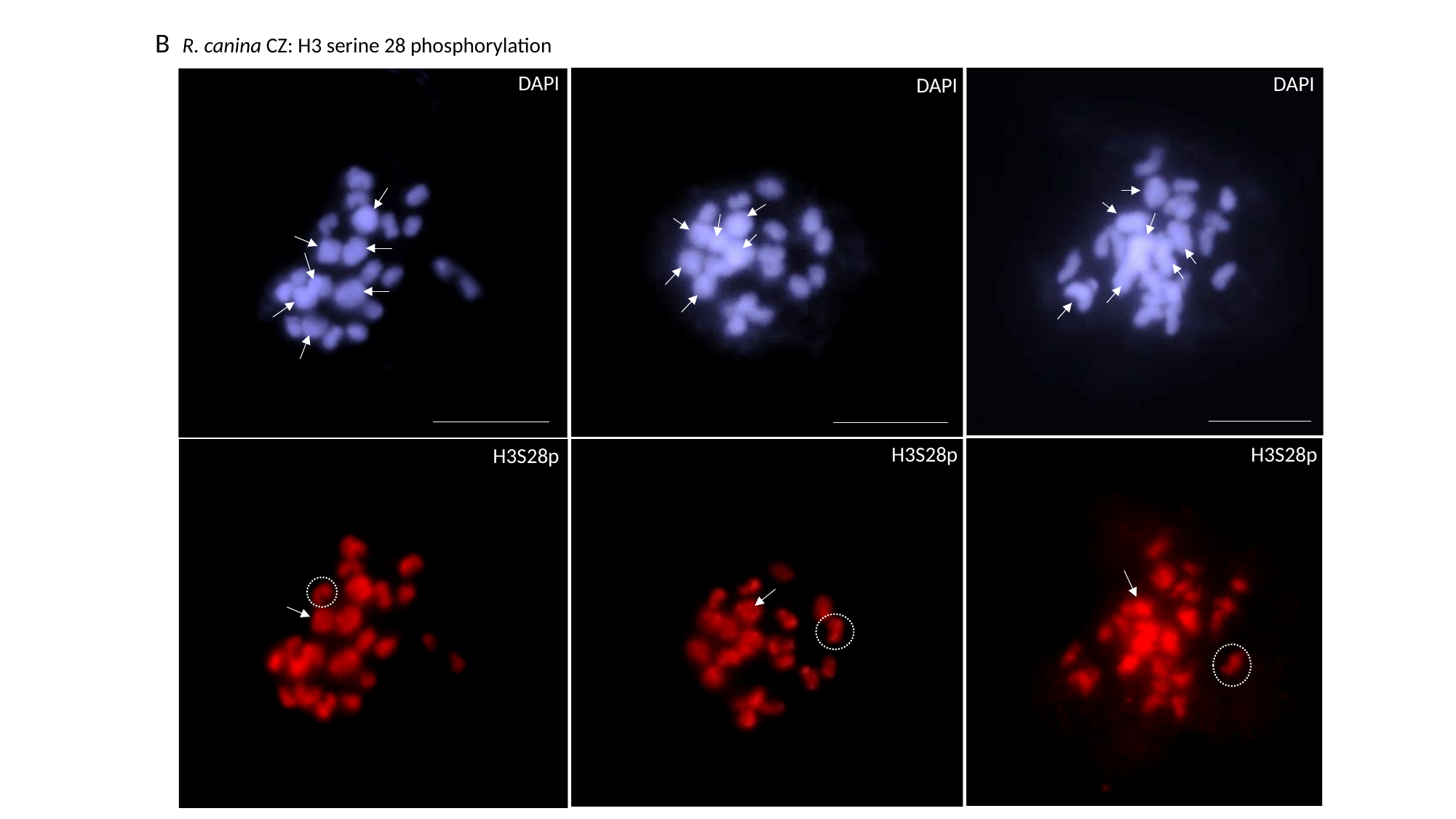

B R. canina CZ: H3 serine 28 phosphorylation
DAPI
DAPI
DAPI
H3S28p
H3S28p
H3S28p

## Slide 3
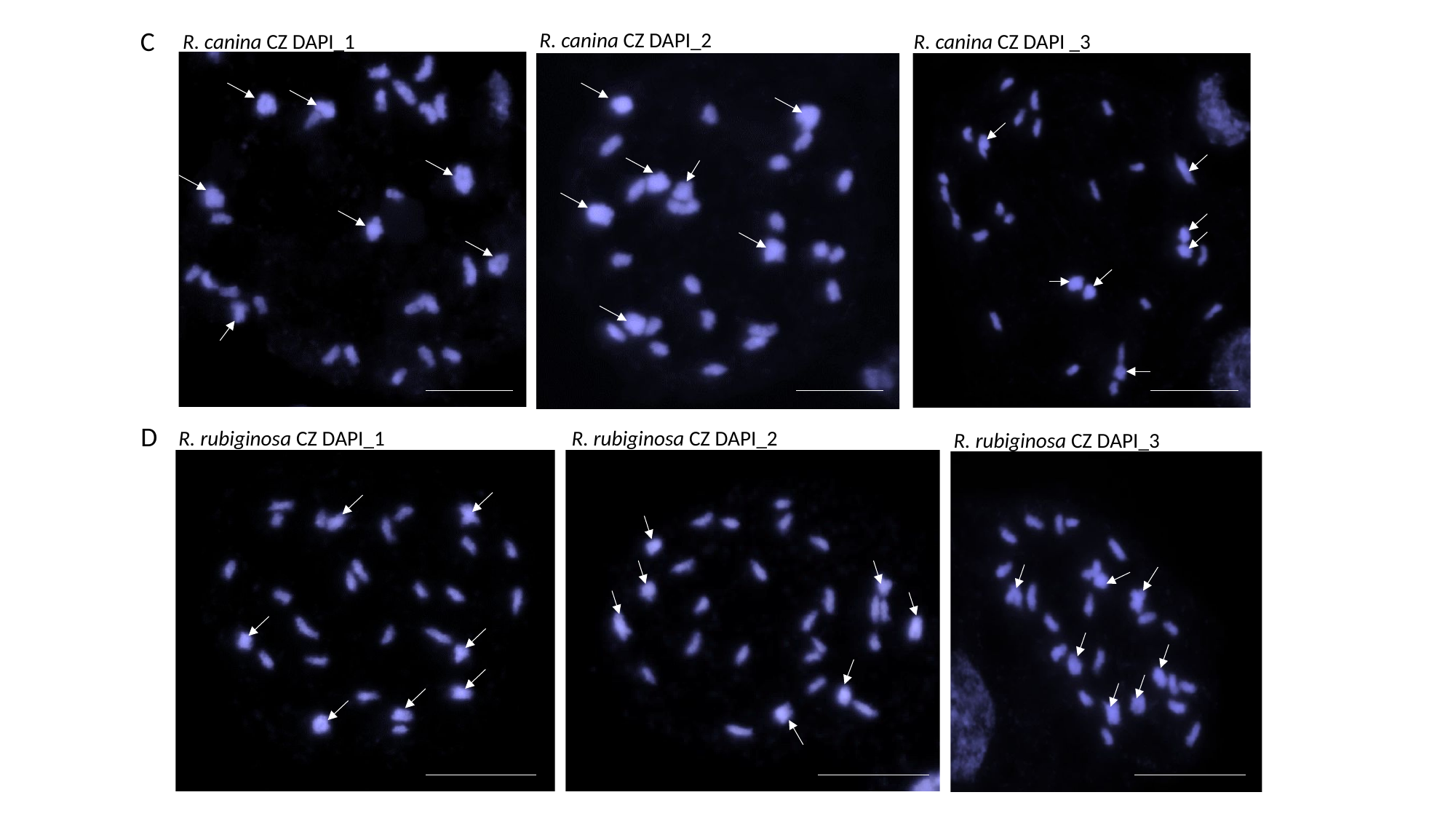

C
R. canina CZ DAPI_2
R. canina CZ DAPI_1
R. canina CZ DAPI _3
D
R. rubiginosa CZ DAPI_1
R. rubiginosa CZ DAPI_2
R. rubiginosa CZ DAPI_3
