# Supplementary material for: Epigenetic histone H3 phosphorylation marks discriminate between univalent- and bivalent-forming chromosomes during canina asymmetrical meiosis
Source: Ann Bot. 2023 Dec 21;133(3):435–46. doi: 10.1093/aob/mcad198 (PMC11006542; doi:10.1093/aob/mcad198)
Supplement: mcad198_suppl_Supplementary_Figures_S2-S3 [file mcad198_suppl_supplementary_figures_s2-s3.pptx]

## Slide 1
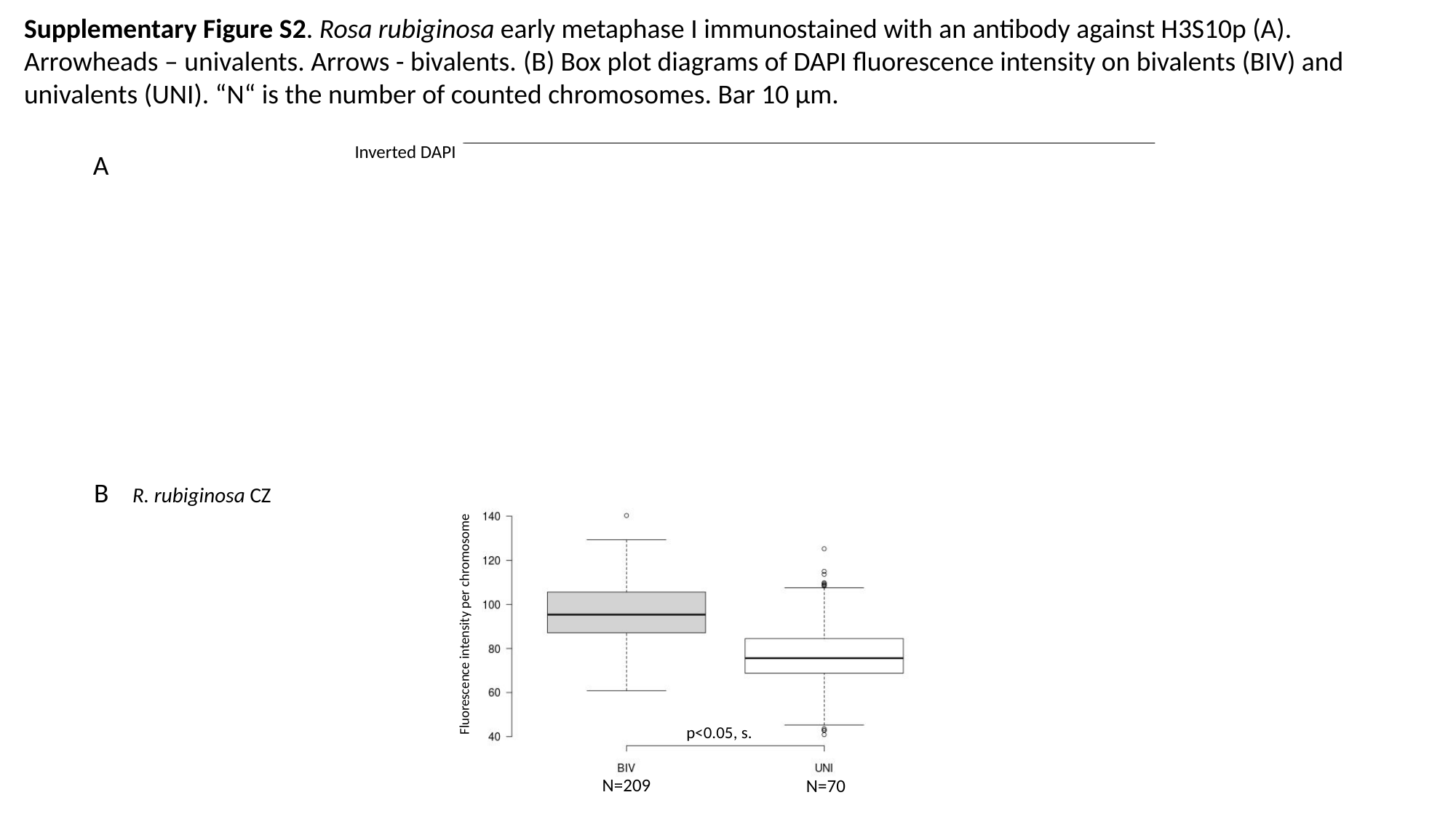

Supplementary Figure S2. Rosa rubiginosa early metaphase I immunostained with an antibody against H3S10p (A). Arrowheads – univalents. Arrows - bivalents. (B) Box plot diagrams of DAPI fluorescence intensity on bivalents (BIV) and univalents (UNI). “N“ is the number of counted chromosomes. Bar 10 µm.
Inverted DAPI
H3S10p
merged
A
B R. rubiginosa CZ
Fluorescence intensity per chromosome
p<0.05, s.
N=209
N=70
