# Supplementary material for: Epigenetic histone H3 phosphorylation marks discriminate between univalent- and bivalent-forming chromosomes during canina asymmetrical meiosis
Source: Ann Bot. 2023 Dec 21;133(3):435–46. doi: 10.1093/aob/mcad198 (PMC11006542; doi:10.1093/aob/mcad198)
Supplement: mcad198_suppl_Supplementary_Figures_S3 [file mcad198_suppl_supplementary_figures_s3.pptx]

## Slide 1
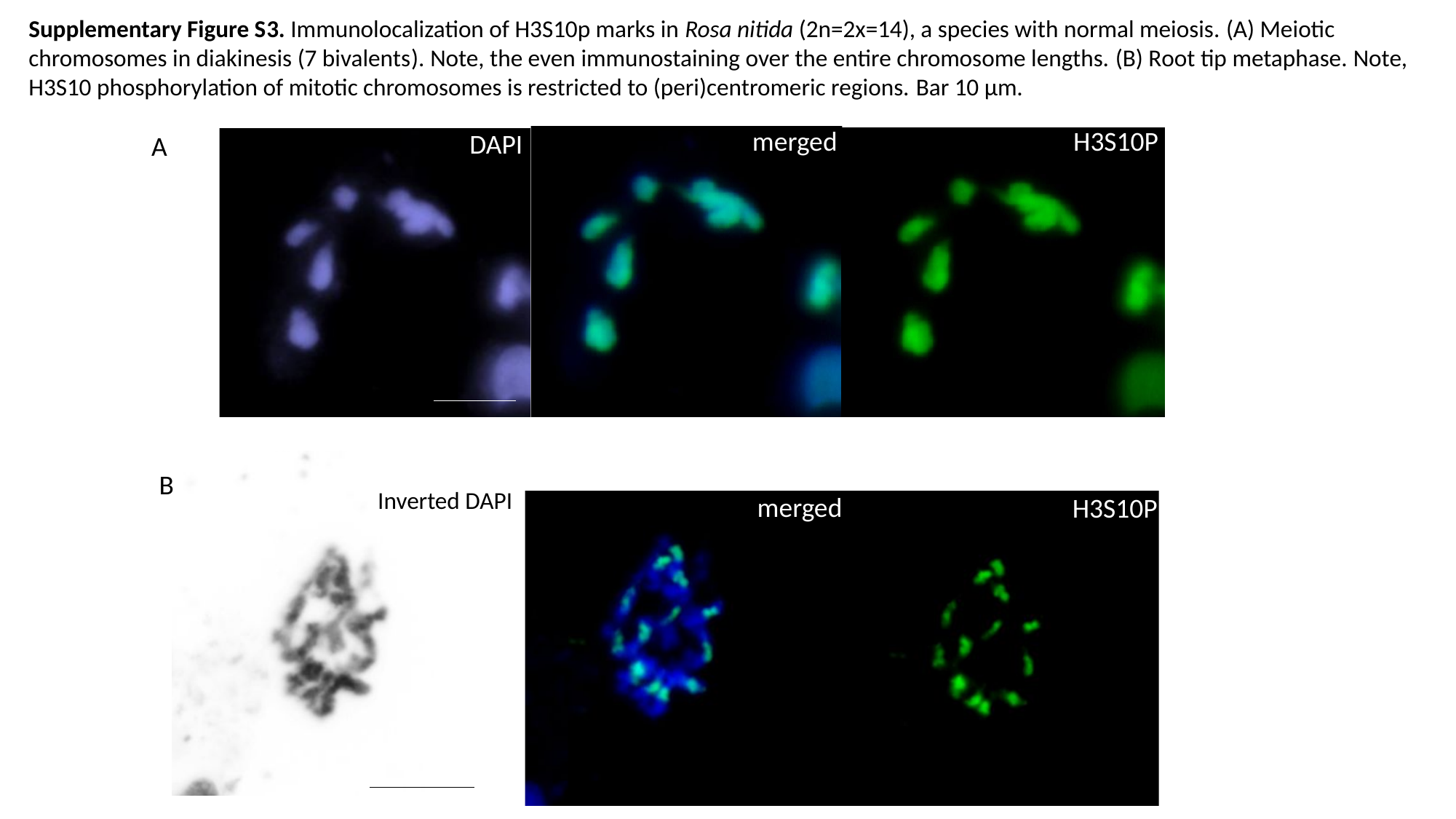

Supplementary Figure S3. Immunolocalization of H3S10p marks in Rosa nitida (2n=2x=14), a species with normal meiosis. (A) Meiotic chromosomes in diakinesis (7 bivalents). Note, the even immunostaining over the entire chromosome lengths. (B) Root tip metaphase. Note, H3S10 phosphorylation of mitotic chromosomes is restricted to (peri)centromeric regions. Bar 10 µm.
merged
H3S10P
DAPI
A
Inverted DAPI
B
merged
H3S10P
