# Supplementary material for: Epigenetic histone H3 phosphorylation marks discriminate between univalent- and bivalent-forming chromosomes during canina asymmetrical meiosis
Source: Ann Bot. 2023 Dec 21;133(3):435–46. doi: 10.1093/aob/mcad198 (PMC11006542; doi:10.1093/aob/mcad198)
Supplement: mcad198_suppl_Supplementary_Figures_S4 [file mcad198_suppl_supplementary_figures_s4.pptx]

## Slide 1
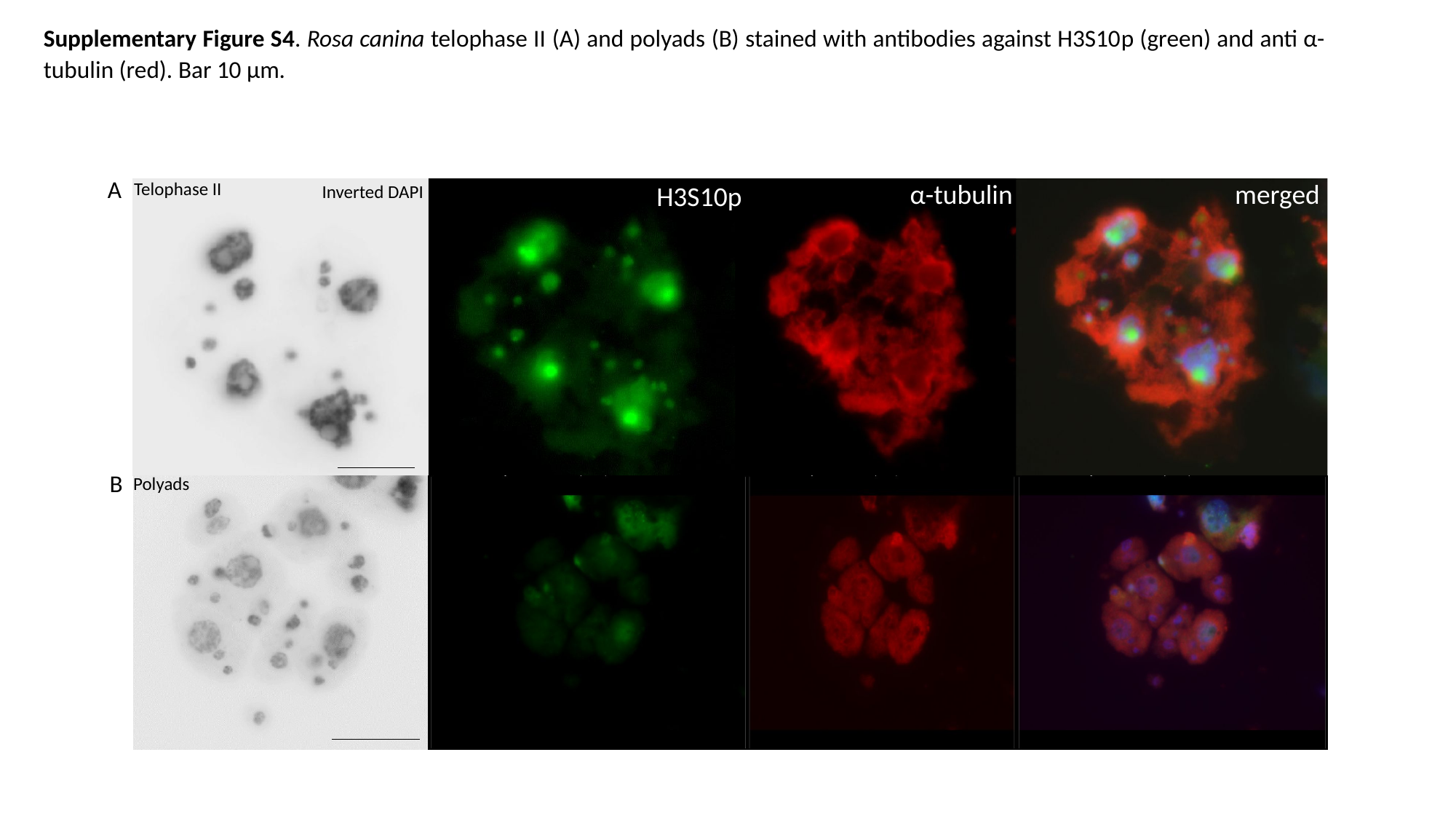

Supplementary Figure S4. Rosa canina telophase II (A) and polyads (B) stained with antibodies against H3S10p (green) and anti α-tubulin (red). Bar 10 µm.
A
Telophase II
B
Polyads
α-tubulin
merged
H3S10p
Inverted DAPI
