# Supplementary material for: Epigenetic histone H3 phosphorylation marks discriminate between univalent- and bivalent-forming chromosomes during canina asymmetrical meiosis
Source: Ann Bot. 2023 Dec 21;133(3):435–46. doi: 10.1093/aob/mcad198 (PMC11006542; doi:10.1093/aob/mcad198)
Supplement: mcad198_suppl_Supplementary_Figures_S5-S6 [file mcad198_suppl_supplementary_figures_s5-s6.pptx]

## Slide 1
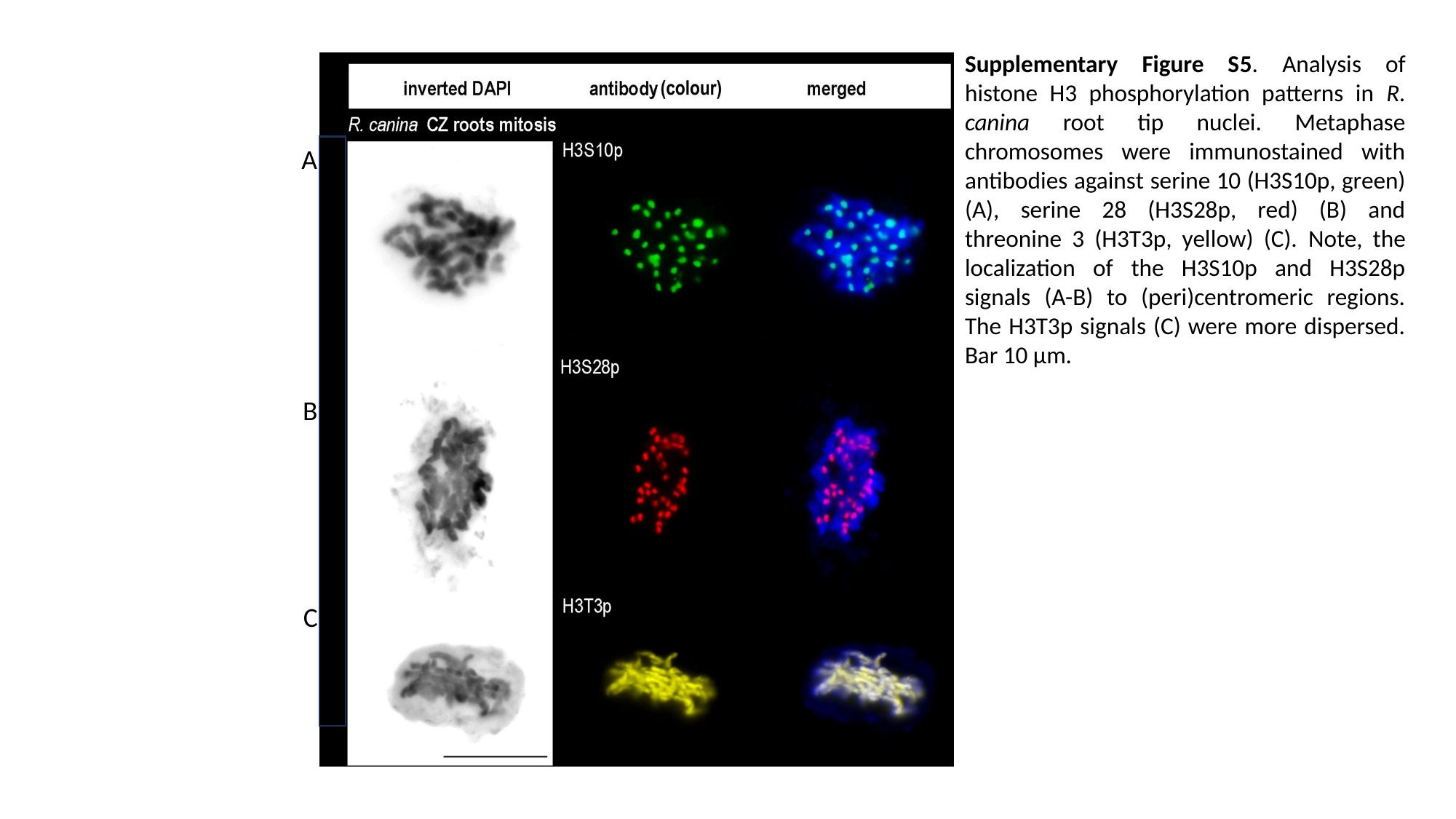

Supplementary Figure S5. Analysis of histone H3 phosphorylation patterns in R. canina root tip nuclei. Metaphase chromosomes were immunostained with antibodies against serine 10 (H3S10p, green) (A), serine 28 (H3S28p, red) (B) and threonine 3 (H3T3p, yellow) (C). Note, the localization of the H3S10p and H3S28p signals (A-B) to (peri)centromeric regions. The H3T3p signals (C) were more dispersed. Bar 10 µm.
A
B
C
