# Supplementary material for: Epigenetic histone H3 phosphorylation marks discriminate between univalent- and bivalent-forming chromosomes during canina asymmetrical meiosis
Source: Ann Bot. 2023 Dec 21;133(3):435–46. doi: 10.1093/aob/mcad198 (PMC11006542; doi:10.1093/aob/mcad198)
Supplement: mcad198_suppl_Supplementary_Figures_S6 [file mcad198_suppl_supplementary_figures_s6.pptx]

## Slide 1
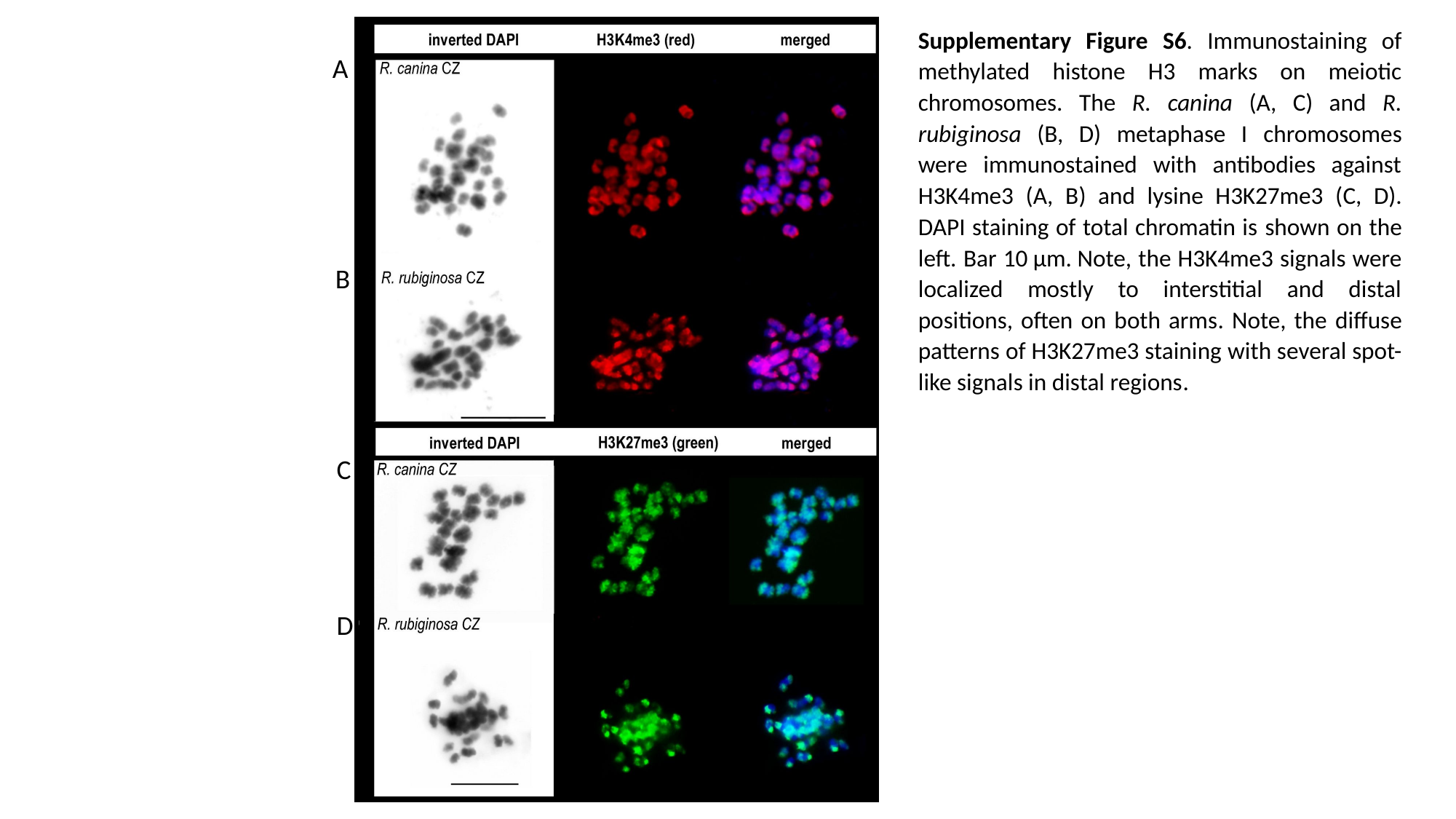

A
B
C
D
Supplementary Figure S6. Immunostaining of methylated histone H3 marks on meiotic chromosomes. The R. canina (A, C) and R. rubiginosa (B, D) metaphase I chromosomes were immunostained with antibodies against H3K4me3 (A, B) and lysine H3K27me3 (C, D). DAPI staining of total chromatin is shown on the left. Bar 10 µm. Note, the H3K4me3 signals were localized mostly to interstitial and distal positions, often on both arms. Note, the diffuse patterns of H3K27me3 staining with several spot-like signals in distal regions.
