# Supplementary material for: Epigenetic histone H3 phosphorylation marks discriminate between univalent- and bivalent-forming chromosomes during canina asymmetrical meiosis
Source: Ann Bot. 2023 Dec 21;133(3):435–46. doi: 10.1093/aob/mcad198 (PMC11006542; doi:10.1093/aob/mcad198)
Supplement: mcad198_suppl_Supplementary_Figures_S7-S8 [file mcad198_suppl_supplementary_figures_s7-s8.pptx]

## Slide 1
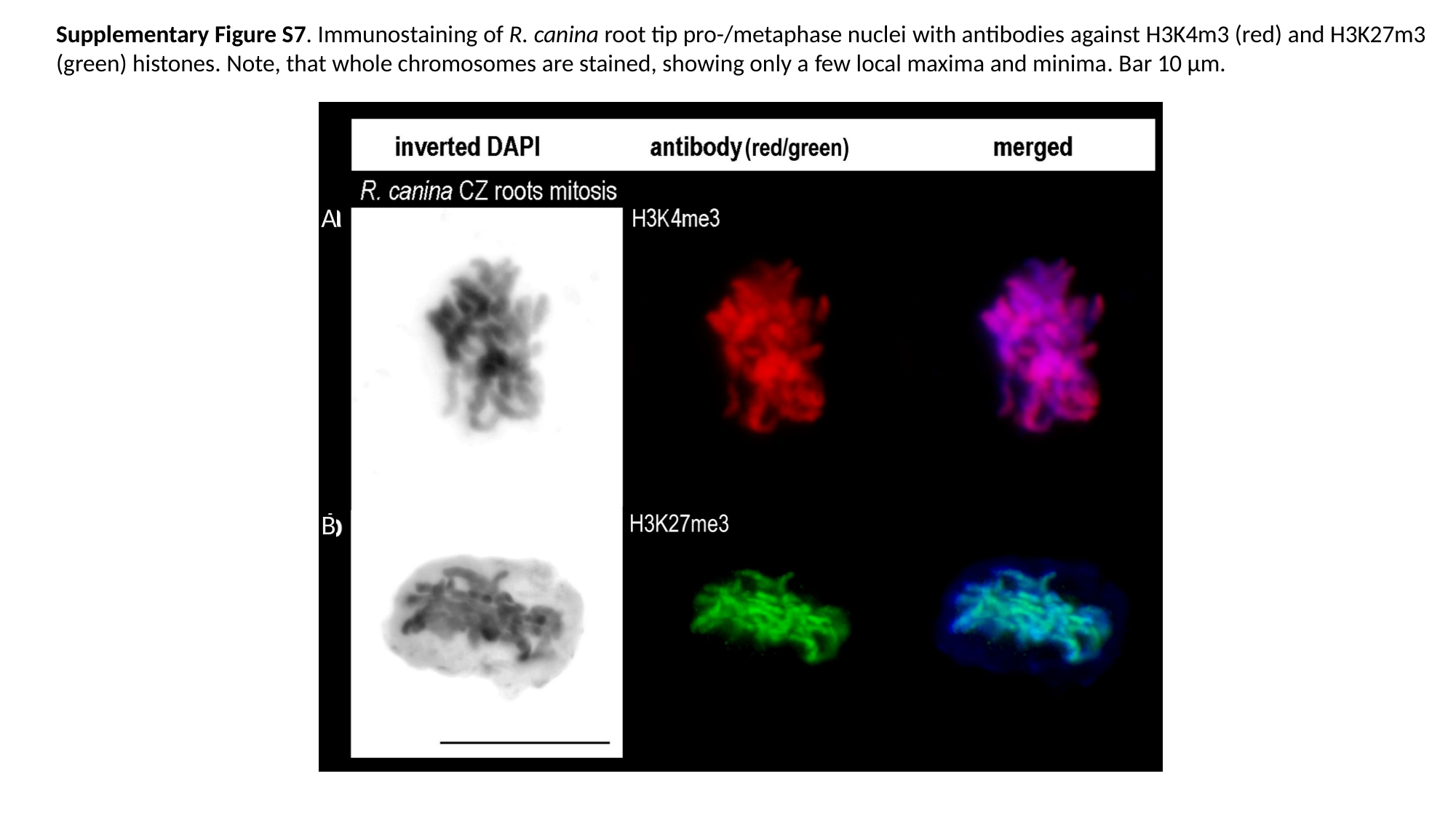

Supplementary Figure S7. Immunostaining of R. canina root tip pro-/metaphase nuclei with antibodies against H3K4m3 (red) and H3K27m3 (green) histones. Note, that whole chromosomes are stained, showing only a few local maxima and minima. Bar 10 µm.
A
B
